# DNA methods for identification of Chinese medicinal materials

**DOI:** 10.1186/1749-8546-2-9

**Published:** 2007-09-05

**Authors:** Pui Ying Yip, Chi Fai Chau, Chun Yin Mak, Hoi Shan Kwan

**Affiliations:** 1 Department of Biology, The Chinese University of Hong Kong, Shatin, Hong Kong; 2 Department of Food Science and Biotechnology, National Chung Hsing University, 250 Kuokuang Road, Taichung 40227, Taiwan

## Abstract

As adulterated and substituted Chinese medicinal materials are common in the market, therapeutic effectiveness of such materials cannot be guaranteed. Identification at species-, strain- and locality-levels, therefore, is required for quality assurance/control of Chinese medicine. This review provides an informative introduction to DNA methods for authentication of Chinese medicinal materials. Technical features and examples of the methods based on sequencing, hybridization and polymerase chain reaction (PCR) are described and their suitability for different identification objectives is discussed.

## Background

Chinese medicinal materials have long been used for disease prevention and therapy in China and are becoming increasingly popular in the West [1-3]. The annual sales of herbal medicines have amounted to US $7 billion in Europe and those in the United States increased from US $200 million in 1988 to more than US $3.3 billion in 1997 [4]. Despite the belief that Chinese medicines are of natural origin which have few adverse effects, there have been numerous reports on adverse effects associated with herbal remedies [5]. One possible cause is the variable quality of both crude medicinal materials (plants, fungi, animal parts and minerals) and Chinese proprietary medicines. Many substitutes and adulterants are in the market due to their lower costs or misidentification caused by similarity in appearance with their authentic counterparts. It is particularly difficult to identify those medicines derived from processed parts of organisms and commercial products in powder and/or tablet forms. Some of the adulterants or substitutes caused intoxications and even deaths [6-8]. Moreover, it is also common for several species to have the same name [9-12]. Inadvertent substitution of these species can also lead to intoxication [13]. Adulterants and substitutes may have completely different or weaker pharmacological actions compared with their authentic counterparts; even different species of the same genus may have totally different actions. For example, *Panax ginseng *(*Renshen*), considered to be 'hot', is used in 'yang-deficient' conditions, while *Panax quinquefolius *(*Xiyangshen*) [14], considered to be 'cool ', is used in 'yin-deficient 'conditions. Authentication of Chinese medicinal materials is the key to ensure the therapeutic potency, minimize unfair trade and raise consumers' confidence towards Chinese medicine in general.

Locality-level identification is also of great importance to ensure highest therapeutic effectiveness. '*Daodi*' is a Chinese term describing the highest quality of herbal materials that are collected from the best region and at the best time [15]. Chinese medicinal materials cultivated in different localities differ in therapeutic effectiveness. For example, it is well accepted that *Atractylodes macrocephala *(*Baizhu*) grown in Jiangning, Jiangshu province, China is more effective than those grown in Zhejiang and Jiangxi provinces [16]. *Condonopsis pilosula *(*Dangshen*) grown in Shanxi province is generally considered to be more potent than those grown in other provinces [16]. Furthermore, samples from the same localities are probably of the same strains; therefore, origin identification helps select the best strains of Chinese medicinal materials. There have been a number of studies investigating medicinal materials grown in different geographical regions. *Codonopsis pilosula *(*Dangshen*) [16], *Panax notoginseng *(*Sanqi*) [15], *Bufo bufo gargarizans *(*Chansu*) [17] and *Paeonia lactiflora *(*Baishao*, or *Chishao*) [18] are just a few examples.

### DNA methods for identification of Chinese medicinal materials

One of the most reliable methods for identification of Chinese medicinal materials is by analyzing DNA that is present in all organisms. DNA methods are suitable for identifying Chinese medicinal materials because genetic composition is unique for each individual irrespective of the physical forms of samples, and is less affected by age [19], physiological conditions, environmental factors [20-22], harvest [19], storage and processing [23-31]. DNA extracted from leaves, stems or roots of a herb all carry the same genetic information [32]. In general, extracted DNA is stable and can be stored at -20°C for a long period of time (about 3–5 years), hence eliminating the time constraint in performing the analysis. A small amount of sample is sufficient for analysis and this is advantageous for analyzing medicinal materials that are expensive or in limited supply [32,33].

In terms of the mechanisms involved, DNA methods can be classified into three types, namely polymerase chain reaction (PCR)-based, hybridization-based and sequencing-based.

#### PCR-based method

PCR-based methods use amplification of the region(s) of interest in the genome; subsequent gel electrophoresis is performed to size and/or score the amplification products.

PCR-based methods have the advantage of requiring tiny amounts of samples for analysis due to the high sensitivity of PCR. However, PCR inhibitors (e.g. polyphenols, pigments and acidic polysaccharides) may be present in DNA samples, thereby hampering amplification. Moreover, PCR is prone to contamination because of its high sensitivity [34]. DNA from contaminating bacteria or fungi in some improperly-stored medicinal samples may be co-amplified if the stringency used is not high enough.

PCR-based methods include sequence characterized amplified regions (SCAR), amplification refractory mutation system (ARMS), simple sequence repeat (SSR) analysis and DNA fingerprinting methods.

##### DNA fingerprinting

DNA fingerprinting refers to simultaneous analysis of multiple loci in a genome to produce a unique pattern for identification. These methods include PCR-restriction fragment length polymorphism (PCR-RFLP), random-primed PCR (RP-PCR), direct amplification of length polymorphism (DALP), inter-simple sequence repeat (ISSR), amplified fragment length polymorphism (AFLP) and directed amplification of minisatellite-region DNA (DAMD). Except PCR-RFLP and DAMD, these methods share the following characteristics:

• Suitable for Chinese medicinal materials which lack DNA sequence information, as they do not require prior sequence knowledge

• Large numbers of loci can be screened in a short time

• Require DNA of good quality, in terms of DNA integrity and the absence of PCR inhibitors

• Unknown origins of the sequences

• Can be used to show the phylogenetic relationships among organisms within the same genus

###### PCR-restriction fragment length polymorphism (PCR-RFLP)

PCR-RFLP uses endonucleases to digest PCR products of regions with sequence polymorphisms. By using an endonuclease which recognizes and cleaves at the polymorphic sites, the digestion of a longer PCR fragment into smaller fragments will change the banding pattern. PCR-RFLP has been used for authentication of *Panax *species [14,35,36], *Fritillaria pallidiflora *[37], *Atractylodes *species [38] and differentiation of *Codonopsis *from their adulterants [39].

Screening PCR products by using various restriction enzymes can be an alternative of sequencing to find out polymorphic regions among samples [34,40]. This method is more reproducible than random priming methods, but it is limited by the degree of polymorphism among individuals within a species [25]. The loss of restriction sites associated with degraded DNA, creation or deletion of restriction sites due to intra-specific variation [34], and the presence of enzyme inhibitors may lead to incomplete digestion.

###### Amplified fragment length polymorphism (AFLP)

AFLP involves restriction of genomic DNA and ligation to adapters, selective amplification of restriction fragments using primers containing the adapter sequences and selective bases at the 3' terminals, and subsequent gel analysis of the amplified fragments [41]. The number of resulting fragments in this multi-locus approach is determined by the number and composition of selective nucleotides as well as the complexity of the genomes. Polymorphisms detected may be caused by a single nucleotide change at the restriction site or 3' end of the primer binding site, insertions and deletions as well as rearrangements. It has been used for authentication [42] and studying genetic diversity [43-46] of Chinese medicines.

AFLP combines the advantages of the reliability of RFLP and the power of PCR. The high reproducibility resulting from stringent reaction conditions enables the use of polymorphic bands in developing cultivar-specific probes, which can then be used for easy identification [47]. By using adapter sequences, fingerprints can be generated without prior sequence knowledge. The number of amplified fragments can be controlled by changing the restriction enzymes and the number of selective bases and thus this method is suitable for DNA of any origin and complexity. It is efficient in revealing polymorphisms even between closely related individuals [48]. The number of polymorphisms per reaction can be higher than RFLP or RAPD [47,49]. Three steps and four different primers are required for analysis of complex genomes [48]. Imperfect ligation or incomplete restriction of DNA will lead to artifactual polymorphisms because of partial fragments [48]. Moreover, if the sequence homology between two organisms is less than 90%, their fingerprints will share very few common fragments [50].

###### Random-primed PCR (RP-PCR)

**Figure 1:**
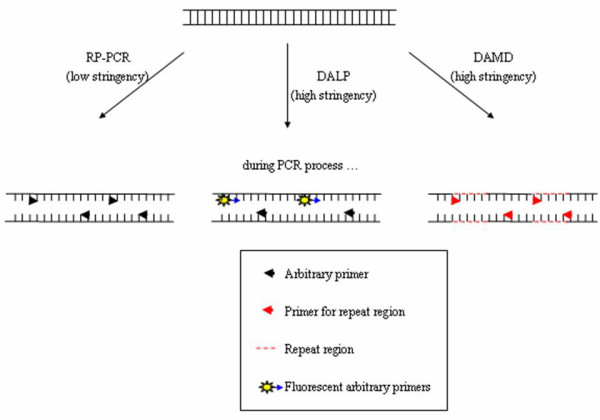
**RP-PCR, DALP and DAMD**. RP-PCR employs low stringency conditions and unlabeled arbitrary primers, while DALP employs high stringency conditions and both fluorescent-labeled and unlabeled arbitrary primers. DAMD employs high stringency conditions and an unlabeled primer targeting repeat regions.

RP-PCR involves amplification at low annealing temperatures using one or two random primers in each PCR reaction to generate unique fingerprints (Figure 1). At reduced stringency, the arbitrary primers bind to a number of sites randomly distributed in the genomic DNA template although the primer and the template sequences may not be perfectly matched. Each anonymous and reproducible fragment is derived from a region of genome that contains, on opposite DNA strands, two primer binding sites located within an amplifiable distance from each other. Polymorphisms are resulted from sequence differences which inhibit primer binding or interfere with amplification. The presence or absence of bands is scored and the results can be used for calculating genetic distances [51-53] and constructing phylogenetic trees [18,54] to study the relationships among samples.

Techniques based on this concept include arbitrarily primed PCR (AP-PCR) [55], random amplified polymorphic DNA (RAPD) [56] and DNA amplification fingerprinting (DAF) [57]. AP-PCR employs primers of approximately 20 nucleotides long and only two relaxed PCR cycles while RAPD employs primers of 10 nucleotides long and all the PCR cycles are relaxed, and DAF employs primers of 5–8 nucleotides long and all the PCR cycles are relaxed with lower stringency than that of RAPD. AP-PCR results are the most reproducible among these three methods.

As any part of the genome, including non-coding regions, may be amplified, these methods can be used to discriminate between closely related individuals. RP-PCR have been used for marker-assisted selection in breeding [58], identification at the individual, variety, strain and species levels [51,59-62], study of genetic diversity [63,64] and differentiation of cultivated and wild samples [18,65,66]. It has been used to authenticate Chinese medicines [51,52,67-69] and identify their geographical origins [16,18,53,70,71].

RP-PCR is a quick and easy method to screen a large number of loci for DNA polymorphisms in a single PCR. Polymorphic markers can be generated rapidly without sequence information. The marker sequences obtained from RP-PCR can also be used to design specific oligonucleotides to be used in SCAR assay [72]. Moreover, any single primer can be used, including those for specific PCR amplification or sequencing. However, there are some limitations in RP-PCR. Firstly, it is sensitive to the reaction conditions, including the amounts of templates [73-75] and magnesium ions [76,77], the sequences of primers [78-81], the presence of glycerol [82] and the quantity and quality of the polymerase [83]. Thermocyclers may also influence the banding patterns due to their different ramp time from the annealing step to the extension step [84,85]. Therefore, results generated with different reaction conditions cannot be compared directly. Secondly, the reproducibility of RP-PCR patterns can be influenced by the quality, quantity and purity of DNA templates [86,87]. Some researchers consider this to have a more significant effect to reproducibility than other factors such as enzyme quality or buffer conditions which may affect only the relative intensities of bands, but do not cause a band to appear or disappear [88]. The number of bands produced can be greatly increased by adding bovine serum albumin (BSA), which prevents various contaminants in impure DNA from binding to *Taq *polymerase [89] thereby stabilizing the polymerase [90]. This method is, therefore, unreliable for identification of medicinal materials with impure or degraded DNA [91] caused by processing or long storage time. Amplification should be performed using DNA templates at two concentrations with at least two-fold difference [92] to ensure reproducibility of the results. Thirdly, as arbitrary primers and low stringencies are used, DNA from any organisms including DNA from contaminants can be amplified, thereby contributing to the banding pattern. Fourthly, as different loci in the genome have different degree of homology among samples (e.g. coding regions are more conserved), the number of loci to be included should be large enough to reveal differences in the whole genome. Fifthly, fragments of the same size in the fingerprints are not necessarily the same sequence [93]. It should not be assumed that bands of a similar size are homologous sequences, especially when distant species are being examined. The extent of polymorphism is indicated by the presence and absence of bands of particular sizes and thus applying RP-PCR on high taxonomic levels leads to an increase in variance of genetic distance estimates.

###### Direct amplification of length polymorphism (DALP)

**Figure 2:**
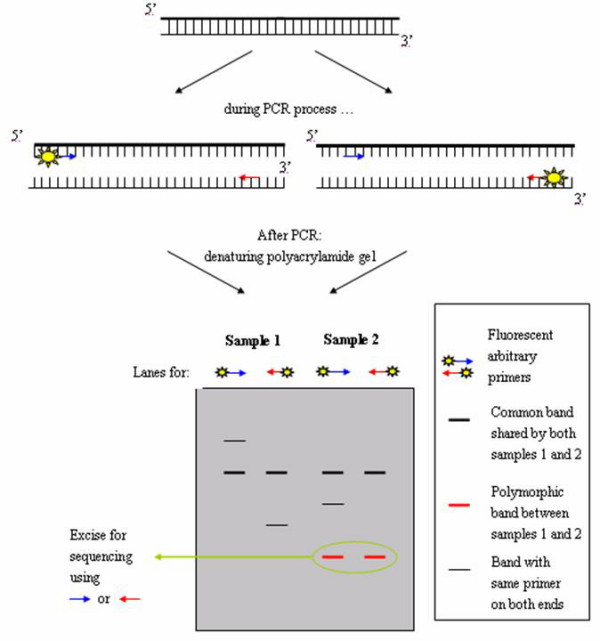
**DALP**. DALP employs two separate PCRs, each containing one of the primers labeled, for each sample. After separation in denaturing polyacrylamide gel, bands shared by the two reactions are products with different primers at both ends. These bands are compared with those of other samples in order to find the polymorphic bands among the samples. Polymorphic bands can be excised from the gel and sequenced directly using either of the primers.

DALP uses a selective forward primer containing a 5' core sequence (e.g. M13 universal sequencing primer) plus additional bases at the 3' end and a common reverse primer (e.g. M13 reverse primer) to generate multibanded patterns in denaturing polyacrylamide gel [48]. For identification of fragments having two different ends, two PCR reactions are performed for each sample, with each reaction containing either of the primers labeled. The common bands in both labeled reactions are the products having two different ends. Any of these bands can be excised from the gel and sequenced directly using forward or reverse primers. After sequencing the polymorphic bands among the samples, species- or strain-specific primers can be designed. These specific primers can then be used for mono-locus amplification (i.e. sequence-tagged site) (Figure 2). DALP is similar to AP-PCR except that it uses higher stringency (i.e. higher annealing temperature, lower concentration of magnesium ion and fewer cycles). At this stringency, a given arbitrary primer will anneal to fixed sites across experiments [48]. Moreover, the polymorphic bands can be sequenced directly to further design species- or strain-specific primers, whereas in AP-PCR the two ends of the products contain the same primer sequence. For this reason, AP-PCR products cannot be sequenced directly (Figure 1). DALP has been used to detect polymorphisms between species [48,94] and between strains [48] and to authenticate *Panax ginseng *(*Renshen*) and *Panax quinquefolius *(*Xiyangshen*) [95]. It has also been used in mapping for marker-assisted selection in breeding [96].

Identification of polymorphic products can be done easily by searching in public sequence databases. However, each sample must be subject to two separate PCR reactions by alternative labeling of the two primers. This requires an additional step apart from identifying polymorphic bands among samples. Unlike RP-PCR, much time and effort are required in screening for suitable primer pairs and optimizing primer ratio [97].

###### Inter-simple sequence repeats (ISSR)

**Figure 3:**
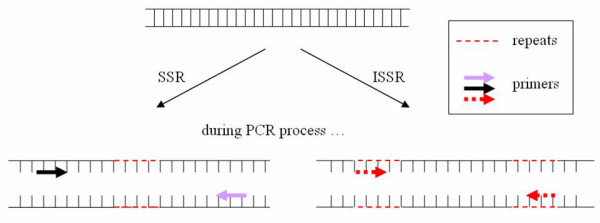
**SSR and ISSR**. SSR employs primers targeting a single repeat region, while ISSR employs a single primer containing repeats to amplify regions between two repeats.

ISSR [98] employs a primer containing simple repeat sequences for PCR amplification to generate fingerprints (Figure 3). The primer can be 5' or 3' anchored by selective nucleotides to prevent internal priming of the primer and to amplify only a subset of the targeted inter-repeat regions, thereby reducing the number of bands produced by priming of dinucleotide inter-repeat region [98-100]. Therefore regions between inversely oriented closely spaced microsatellites are amplified [101]. This method relies on the existence of 'SSR hot spots' in genomes [98,101]. It has been used in the authentication of *Dendrobium officinale *(*Tiepi Shihu*) [102] and in the studies of genetic variations and relationships [103-106].

As simple sequence repeats exist in any genome, this method allows fingerprints to be generated for any organism. However, the primers may anneal to sequences other than microsatellites of the desired repeats as in RAPD [99]. Moreover, the banding patterns can be affected by magnesium ion concentration, thermocycler and annealing temperature in use [102]. Compared to SSR markers, ISSR markers are not locus-specific and anonymous bands are produced in the fingerprints [101].

###### Directed amplification of minisatellite-region DNA (DAMD)

Minisatellites are also known as variable number of tandem repeats (VNTR). They are similar to microsatellites except that the repeat unit sequence is longer than 10 bp (the distinction between microsatellites and minisatellites is often arbitrary with repeat units between 8 and 15 bp [107]). DAMD [108] is a DNA fingerprinting method based on amplification of the regions rich in minisatellites at relatively high stringencies by using previously found VNTR core sequences as primers (Figure 1). It is employed in identification of species-specific sequences [109]. The method has been used for authentication of *Panax ginseng *(*Renshen*) and *Panax quinquefolius *(*Xiyangshen*) [42] and characterization of varieties [78] and cell lines [110].

Similar to SSR, minisatellites are present in all organisms, making it possible to apply this method to any genome. Examples include plants such as mulberry [78] and oilseed [109], mushrooms *Agaricus bisporus *and *Pleurotus *[111], animals such as human, snake and mouse [110], insects such as mosquito and moth. This method is more reproducible than RAPD due to the longer primers used [111]. However, similar to ISSR, it only reveals polymorphisms in the regions rich in repetitive sequences, while RP-PCR can represent the polymorphisms of the whole genome [78]. Moreover, polymorphic sequence must be obtained by cloning polymorphic bands prior to sequencing because of the same primer sequences at both ends of the PCR product.

##### Sequence characterized amplified regions (SCAR)

SCAR [72] can be used for detection or differentiation of samples by using specific primers designed from polymorphic RAPD [72,112] or ISSR [113,114] fragments for PCR, leading to positive or negative amplification in target-containing and non-target-containing samples respectively [112,115] or amplification products of different sizes in the case of closely related samples [91,116]. This method has been used for authentication of *Panax *[91] and crocodilian species [115], and for discrimination of *Artemisia princeps *(*Kuihao*) and *Artemisia argyi *(*Aiye*) from other *Artemisia *herbs [112].

SCAR markers are more reproducible than RAPD markers, and they are straightforward for data interpretation. As little DNA is required for PCR, DNA extraction must be performed on a sample representative of the mix in order to accurately detect a target in a mixture. Prior sequence information (i.e. sequencing the polymorphic fragments) is required for designing the primers flanking the polymorphic region. As PCR inhibitory effects of ingredients in Chinese medicine can lead to false negative results, amplification of a control fragment using the same DNA template [114,115] or spiking control DNA amplifiable by the same primers to the sample DNA should be performed to ensure that the quality of sample DNA is suitable for PCR.

##### Amplification refractory mutation system (ARMS)

ARMS [117] is also known as allele-specific PCR (AS-PCR) [118]. It refers to PCR amplification using primers which differ in the 3' terminal for distinguishing related samples. This method is based on the fact that mismatches at the last base(s) at the 3' terminal of primers can lead to failure in PCR [119,120]. It has been used to identify *Panax *species [121] and *Curcuma *(*Ezhu*) species [122] using primers based on chloroplast *trn*K and nuclear 18S rRNA genes. ARMS based on mitochondrial *cytochrome b *gene has been used for detection of tiger bone DNA [123]. It has also been used for distinguishing *Myospalax baileyi *[124] and gecko [125] from their substitutes and adulterants.

This method is simple, rapid and reliable [117]. However, as the absence of bands is also considered as a positive result for detection experiments, appropriate positive controls should be included to show that the reagents have indeed worked properly and that the PCR process was not problematic. PCR inhibitory effects leading to false negative results can be identified by parallel testing of samples spiked with tiny quantities of pure target DNA to demonstrate the level of detection [123].

##### Simple sequence repeats (SSR) analysis

SSR analysis is also referred to as simple sequence length polymorphism (SSLP). SSR [126,127] is also known as microsatellites, short tandem repeats and sequence-tagged microsatellite sites (STMS) which are short tandem repeats of 2–8 nucleotides [128,129] or 1–6 nucleotides [130-132] widely and abundantly dispersed in most nuclear eukaryotic genomes [130,133,134]. Changes in repeat numbers at SSR loci are much more frequent than normal mutation rate [101,135,136] because of slippage [133,136-138] and recombination [133,138-140]. The different numbers of repeating units (alleles) in polymorphic loci lead to variation in band sizes (length polymorphism) when specific flanking primers designed based on conserved sequences are used for amplification of the loci in organisms of the same species. The presence or absence of each allele at each locus can be scored digitally. Figure 3 shows the procedural differences between ISSR and SSR.

SSR analysis has been applied in authentication of ginseng [25,141]. In SSR analysis, *Panax quinquefolius *(*Xiyangshen*) showed different allele patterns compared with those of *Panax ginseng *(*Renshen*). Moreover, cultivated and wild *Panax quinquefolius *(*Xiyangshen*) can be distinguished from each other [141]. This method has also been used in characterization of germplasm resource of *Gastrodia elata *(*Tianma*) [142] and studying genomic variation in regenerants of *Codonopsis lanceolata *(*Dangshen*) [104]. Due to its ability for analysis at low taxonomic levels, it has been applied to breeding programs [143].

SSR loci are highly polymorphic [101,144]. In bread wheat, loci with 4 to 40 alleles have been found for 480 varieties [145], with an average of 16.4 alleles. It is possible to multiplex 17 loci in a single PCR reaction [146]. As the primers are designed according to conserved sequences, they may be successfully applied for related species [147,148] and sometimes across genera [147,149]. Moreover, the primers can be accessible to other laboratories via published primer sequences [150-152]. Besides the advantages of employing PCR and the high reproducibility, this technique can be fully automated (including automated sizing of PCR products by fluorescence-based detection) and fitted into large-scale, high throughput authentication centers [25].

Size differences can also be caused by insertions and deletions, and duplication events in the flanking sequences [153-155]. However, high initial investment and technical expertise are required for development of SSR markers [156-158]. Although SSR markers can be identified by screening DNA [150,159,160] or EST sequence databases [161-163], sequence information is not available for most Chinese medicinal species. Without sequence information, SSR marker development involves either constructing enriched genomic libraries [142,147,149,164], ISSR genomic pools [165,166] or Southern hybridization-screened RAPD genomic pools [167,168] followed by cloning and sequencing. However, the identified loci may not be polymorphic or informative [169]. Moreover, null alleles (i.e. loss of PCR products occurring due to mutations in the primer binding sites) may be encountered [143,150,158] even when using SSR markers developed from the same species [170].

#### Sequencing-based methods

Much information can be obtained from DNA sequences [34]. DNA sequences can be used for studying phylogenetic relationships among different species [70,171,172]. Sequencing also allows detection of new or unusual species [34,173]. Another advantage of using sequencing for species identification is that the identities of adulterants can be identified by performing sequence searches on public sequence databases such as GenBank. By database searching, Mihalov *et al*. [174] successfully identified soybean substituted for ginseng (*Panax *species) in commercial samples. However, prior sequence knowledge is required for designing primers for amplification of the region of interest [34].

DNA sequencing can be used to assess variations due to transversions, transitions, insertions or deletions. Different regions in the genome evolve at different rates, which are useful for identification at different taxonomic levels [33]. Regions that do not code for proteins are under lower selective pressure and thus can be more variable. The more variable is a particular region, the more closely related individuals can be differentiated by this region. Regions commonly used include rRNA genes, mitochondrial genes and chloroplast genes [175]. There are more than 100 copies of these genes in a cell, which evolve inter-dependently (i.e. concerted evolution [176-178], previously known as horizontal evolution [179] in the case of rRNA genes, and homoplasmy [180,181] in the cases of mitochondrial and chloroplast genes). Thus, methods using these regions are highly sensitive and amplifications are facilitated. This is also advantageous during PCR amplification of medicinal samples with degraded DNA because the presence of one full-length copy in a reaction is theoretically enough for amplification. Moreover, the conserved sequences flanking the genes are useful for designing 'universal' primers to be used for amplifying and sequencing the genes from many species [182-185].

rDNA sequences have been used for studying Chinese medicines such as *Panax *species [35,186-188], *Eucommia ulmoides *(*Duzhong*) [189], *Cordyceps *(*Dongchongxiacao*) species [190], *Dendrobium *(*Shihu*) species [191], *Myospalax baileyi *[192] and *Ligusticum chuanxiong *(*Chuanxiong*) [193]. The chloroplast sequences have been used for studying *Curcuma *(*Ezhu*) species [194], *Panax notoginseng *(*Sanqi*) [67,188], *Adenophora *(*Shashen*) species [195] and *Atractylodes *(*Cangzhu*) species [38]. The mitochondrial sequences have been used for studying *Bungarus parvus *[196], *Hippocampus *species [197] and turtle shells [198].

#### Hybridization-based methods

Nucleic acid hybridization is a process in which two complementary single-stranded nucleic acids anneal into a double-stranded nucleic acid through the formation of hydrogen bonds. DNA hybridization has been used for detection of *Dendrobium *(*Shihu*) species by incorporating a species-specific region of reference samples on a glass slide and subsequent hybridization of the region amplified from a complex mixture of medicinal materials using universal primers [199]. Besides *Dendrobium *(*Shihu*) [200], it has also been used to identify *Fritillaria *(*Chuanbeimu*) species [201] and toxic Chinese medicinal materials such as *Datura *(*Yangjinhua*) species [202,203], *Pinellia *(*Banxia*) species [33,202] and others[202,204].

With DNA hybridization, detection from a variety of possible species is feasible [204]. Detection of the presence of multiple species in admixtures [205,206] and in a wide range of commercially processed, heated and canned food products have also been demonstrated [206-208]. Moreover, DNA hybridization has been used to identify organisms of different breeds [209]. If the probes are oligonucleotides shorter than 100 bases, hybridization is possible even after a considerable level of DNA degradation [34]. However, a relatively large amount of DNA is required and the process is time-consuming (because the hybridization step typically requires overnight incubation) [34] and labor-intensive compared to PCR-based methods. If the experimental conditions are not stringent enough, cross-hybridization with highly similar but not identical targets may also occur [204,210,211], resulting in false-positive results. Furthermore, only a limited number of probes can be applied in one hybridization experiment [212,213] for most hybridization methods. As a result, much time has to be spent on testing a large number of probes and checking for false-positives and false-negatives. Despite the fact that microarray hybridization has a high throughput, it is still quite expensive.

### Comparison of methods

#### Level of identification

The level of identification by a certain method largely depends on its targeted region in the genome and its mechanism and principle of analysis. Species-specific regions, such as internal transcribed spacers (ITS) and 5S rRNA spacer regions of ribosomal DNA, mitochondrial *cytochrome b *and chloroplast *trn*L-*trn*F spacer regions, have been used for species-level identification by sequencing [53,70,174], microarray hybridization [199,204] and PCR [123]. SCAR, in which the species-specific bands generated from fingerprints are used for primer construction [91,112], is applicable to species-level identification. However, techniques based on differences of several bases, such as ARMS and PCR-RFLP, can be applied to species-level identification or lower even when more conserved genes are employed, as long as sufficient polymorphic nucleotides can be found in the genes. For example, 18S ribosomal gene has been used for PCR-RFLP for differentiation of *Panax ginseng *(*Renshen*) from 4 localities [36]. The chloroplast *trn*K gene and the nuclear 18S rRNA gene have been used for ARMS to identify *Panax *species [121] and *Curcuma *(*Ezhu*) species [122].

Except for PCR-RFLP, fingerprinting methods can only be used to discriminate among closely related individuals, mostly on population [214,215], strain and locality [36,53,59,62] levels. However, if scoring of bands for further processing is not required, fingerprinting methods can be applied in differentiation at the species level [51,60] by banding pattern comparison with reference materials.

SSR analysis can be used in identification of species within the same genus [25,141], at the variety and cultivar level [143,145,216] as well as at the individual level [217,218]. It is not used for comparing species of different genus because the sequences of the amplification products can be quite different even if the primers can successfully amplify PCR products [153,219]. ISSR involves amplification of regions between SSR loci and can be used for authentication at population [102,220,221] and species levels [222,223].

#### Choice of method

**Figure 4:**
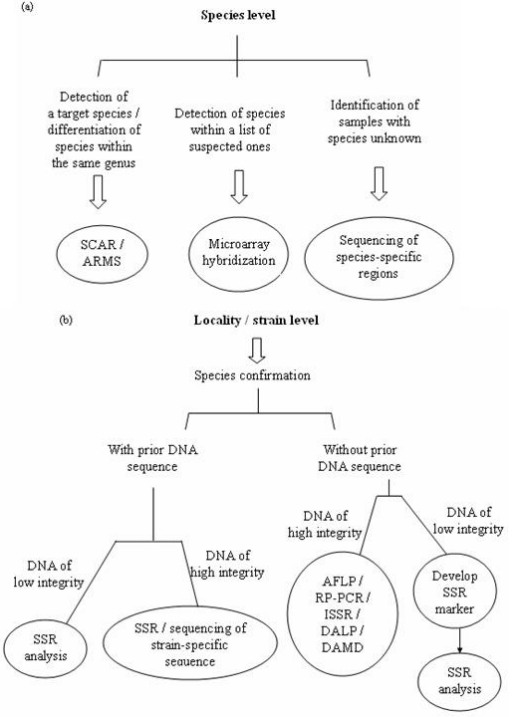
**Selection of appropriate molecular identification methods for (a) species and (b) locality/strain levels**. a) For species level, SCAR and ARMS are suitable for detection of a target species or differentiation of species within the same genus. Microarray hybridization is suitable for detection of species within a list of suspected ones. Sequencing of species-specific regions is suitable for identification of samples with its species totally unknown. b) For locality or strain level, species identification should be performed first. Then, depending on whether prior sequence knowledge is available and the integrity of the DNA samples, different methods can be applied. SSR analysis and sequencing of strain-specific sequences are suitable for samples with primer sequences already available and SSR analysis is especially suitable for samples with DNA of low integrity. For samples without prior sequence knowledge, SSR marker development followed by subsequent SSR analysis can be applied for DNA samples of low integrity, while AFLP, RP-PCR, ISSR, DALP and DAMD can be applied for DNA samples of high integrity.

**Figure 5:**
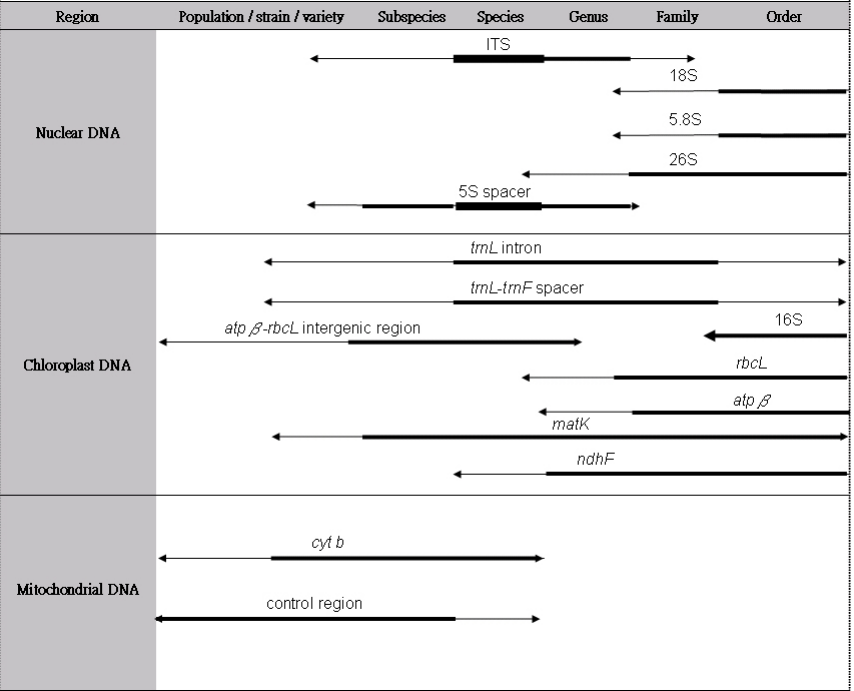
**Comparison of various regions for use on various taxonomic levels (adapted from [244-246])**. The thickness of the lines represents how frequently the regions are used for identification at the levels. The exact taxonomic levels that the regions can be applied to depend on the selected method. Successful application on a species does not guarantee successful application on other species at the same taxonomic level. For nuclear DNA regions, 18S and 5.8S regions are usually used for identification to the order or family levels and 26S is usually used for the order level down to the species level. The two ITS regions and the 5S spacer region are commonly used for identification at the species level, but they can also be applied down to the population/strain/variety level. For chloroplast DNA regions, 16S is suitable for the order level, while *rbcL*, *atpβ *and *ndhF *are suitable for the levels from order to species. The *trnL *intron, *trnL*-*trnF *spacer and *matK *regions can be applied to the levels from order to population/strain/variety, but the former two are more commonly used for the levels from family to species, while the latter is commonly used for the levels from order to subspecies. The *atpβ*-*rbcL *region can be used for the levels from genus to population/strain/variety, but it is more commonly used for the levels from genus to subspecies. For mitochondrial regions, *cytochrome b *and the control region can be used for identification at the levels from species to population/strain/variety, but the former is more commonly used for the species level and the latter is more commonly used for the subspecies level and lower.

Table 1 summarizes the methods mentioned in this article. Each method has its own advantages and limitations. Decisions as to which method to use should be based on the aim, the DNA quality of the obtained sample and the cost of implementation (e.g. whether prior sequence knowledge is obtainable and required). A diagram showing the technical considerations affecting the choice of method is provided in Figure 4. Besides method selection, choosing a suitable DNA region for analysis is also crucial in obtaining good results (Figure 5). It should be noted that the exact taxonomic levels the regions can be applied to depend on the choice of method, and that successful application on a species does not guarantee successful application on other species at the same taxonomic level.

**Table 1:** Comparison of various DNA methods (adapted from [135, 237, 245, 247])

	**SCAR**	**ARMS**	**SSR**	**PCR-RFLP**	**RP-PCR**	**ISSR**	**AFLP**	**Sequencing**	**Hybridization**
**Development cost**	medium	medium	high	medium ^a^	low	low	low	medium	medium
**Running cost**	low	low	medium	medium ^a^	low	low	medium	medium	medium
**Polymorphism**	low ^b^	low ^c^	high ^d^	low ^e^	medium ^f^	medium ^f^	medium ^f^	medium-low ^g^	only for detection ^h^
**Detection of contamination by DNA of the same target species (mixture)**	no	no	yes	yes/no^i^	no	no	no	yes/no^k^	yes/no^k^
**Detection of contamination by non-target species DNA (mixture)**	no	no	no	yes ^j^	no	no	no	yes/no^k^	yes/no^k^
**Detection of contamination by DNA of the same target species (mixture)**	no	no	yes	yes/no ^i^	no	no	no	yes/no^k^	yes/no^k^
**Detection of contamination by non-target species DNA (mixture)**	no	No	no	yes^j^	No	no	no	yes/no^k^	yes/no^k^
**Multi-locus/single locus**	single locus	single locus	single locus	single locus	multi-loci	multi-loci	multi-loci	single locus	single locus
**Level of identification (taxonomic level)**	species	species to strain	strain	species to strain	species to strain	species to strain	species to strain	family to strain	genus to subspecies
**Quality of DNA required (integrity)**	medium	medium	low	medium	high	high	high	medium	medium
**Quality of DNA required (purity)**	high ^l^	high ^l^	medium	high	high	high	high	high ^l^	high
**Prior sequence knowledge requirement (including universal primer sequence)**	yes	yes	yes	no	no	no	no	yes	yes
**Throughput**	low	low	high	low	high	high	high	low	high
**Level of skills required**	low	low	low-medium	low	low	low	medium	medium	medium
**Automation**	yes	yes	yes	difficult	yes	yes	yes	yes	difficult
**Reliability**	high	high	high	high	low	medium	high	high	medium

**Development cost**: cost for developing standardized reference results for target samples

**Running cost**: cost for routine testing

**Detection of contamination**: same target species = samples of the same strain or locality; non-target species = adulterants or substitutes

**Multi-locus**: simultaneous amplification of multiple regions in the genome in a single PCR

**Prior sequence requirement**: sequence requirement to design primers for amplification or probes for hybridization

**Throughput**: number of samples that can be handled within a given period of time

**Reliability**: giving reproducible and accurate results under the same conditions

a: enzyme dependent

b: band/no band, or size polymorphism

c: band or no band

d: null allele or size polymorphism

e: band number and size polymorphism

f: presence/absence of all the bands in a pattern

g: sequence dependent; nucleotide difference

h: presence or absence of sample DNA

i: restriction enzyme dependent

j: primer dependent

k: sequence dependent

l: crude tolerated

### Problems faced for using DNA in identification

While DNA methods are excellent for identifying Chinese medicinal materials of various forms, certain problems remain to be solved. DNA degradation is common for those materials that have been heat-treated [123,224-226] by frying, sun-drying, oven-drying or milling without cooling, treated with various chemicals and stored for a long time in various conditions. This problem may be overcome by choosing more degradation-tolerant methods, one of which is employing multi-copy genes (e.g. ribosomal and mitochondrial genes) [227] so that the chances of getting some full-length copies are higher. The mitochondrial genes are also suitable because mitochondria are relatively intact during processing [228]. Another approach is to analyze small regions of DNA by methods such as SSR analysis [229,230], which may target sequences as short as several tens of base pairs of DNA and the chances of breakage within the target region are minimal [231-233]. Hybridization is also useful in identification of degraded DNA [206,207].

The presence of PCR inhibitors is a problem. Chinese medicinal materials are usually (1) plants that may contain phenolic compounds, acidic polysaccharides and pigments, (2) fungi which may contain acidic polysaccharides and pigments, or (3) animal remains which may contain fat and complex polysaccharides. Choosing the most suitable DNA extraction procedures for the types of samples may help eliminate the PCR inhibitors [234-237]. Other possible solutions are diluting the extracted DNA and adding PCR enhancers.

Contamination by non-target DNA of bacteria, fungi or insects due to improper storage or by other medicinal materials in formulations is another challenge. This creates the biggest problem for fingerprinting methods because the DNA of the contaminants will be amplified. This may be overcome by using methods specific for the target such as ARMS and SCAR. If the region of analysis is species-specific and the stringency is high enough, microarray analysis is also an alternative for studying DNA mixtures [199,238]. However, DNA degradation by microbes cannot be avoided in contaminated samples [239].

DNA information is not directly correlated with the amounts of active ingredients. However, genetic data have its own advantages (as discussed above) over other methods for authentication and identification.

There are successful cases of using DNA methods to identify Chinese medicines, such as *Panax *[174,186,240] (Table 2), crocodilian species [115] and *Dendrobium *(*Shihu*) species [200,241] in commercial products. DNA methods can also be used to identify components in concentrated Chinese medicine preparations in which the components have been grounded, boiled, filtrated, concentrated, dried and blended [242]. By applying appropriate methods and regions of genome, problems in authentication of Chinese medicinal materials can be solved.

**Table 2:** DNA methods for identification of *Panax *species

**Techniques**	**Samples to differentiate**	**Prerequisite**	**Summary of results**	**Year of publication**	**Ref**
PCR-RFLP	Six *Panax *species and adulterants	ITS1-5.8S-ITS2 sequences	The polymorphisms in the ITS1-5.8S-ITS2 sequence among the samples can be shown using various restriction enzymes to create species-specific RFLP profiles. Ten per cent of contamination of *P. ginseng *was detected from *P. quinquefolius *sample.	1999	14
PCR-RFLP	Three *Panax *species	18S sequences	Based on the 18S rRNA gene, three *Panax *species gave species-specific electrophoretic profiles by PCR-RFLP and expected fragments corresponding to each species were detected only under defined conditions by mutant allele specific amplification (MASA, i.e. ARMS) analyses.	1997	35
PCR-RFLP	*P. ginseng *from four localities, one from China and three from Korea	18S sequences	A ginseng sample showed fragments distinctive from the other two samples of the same country but different locality origins.	2001	36
AFLP	*P. ginseng *and *P. quinquefolius*	no	Polymorphic bands unique to *P. ginseng *and to *P. quinquefolius *and bands common to them were identified. *P. ginseng *samples from different farms in China and Korea are homogeneous genetically. *P. quinquefolius *from different sources are much more heterogeneous.	2002	42
RAPD	*P. ginseng *from four localities, one from China and three from Korea	no	Similarity among the DNA of ginseng plants analyzed was low.	2001	36
RAPD	Three strains of *P. ginseng*	no	A 725 bp band was present in the elite strain Aizu K-111 (now called *Kaishusan*) while the other strains did not always show this band.	2001	62
RAPD	Two cultivated groups of ginseng	no	Cluster analysis of the patterns showed that the genetic relationship between different strains of Da-maya group is closer than those strains of E-maya group.	2004	64
RAPD and AP-PCR	Three *Panax *species and adulterants	no	Fingerprints for *P. ginseng *or *P. quinquefolius *were basically consistent irrespective of plant source or age compared to the very different fingerprints from adulterants. The degree of similarity of the fingerprints confirmed that *P. ginseng *is more closely related to *P. quinquefolius *than it is to *P. notoginseng*.	1995	52
AP-PCR	Oriental ginseng from two sources and two American ginseng	no	Oriental ginseng from two sources produced nearly identical fingerprints. Two American ginseng samples gave fingerprints distinctive to the species with two primers tested but polymorphic and significantly different fingerprints with one of the primers tested. Therefore, the American ginseng samples were supposed to be from different strains.	1994	60
DALP	*P. ginseng *and *P. quinquefolius*	no	A DALP fragment was found present in all *P. ginsengs *but absent in all the *P. quinquefolius *examined. The species-specific band was converted to sequence-tagged site (i.e. amplification by specific primers giving a species-specific band) for quick authentication.	2001	95
DAMD	*P. ginseng *and *P. quinquefolius*	polymorphic AFLP band	A polymorphic AFLP band in *P. ginseng *contains a minisatellite which can be used in DAMD analysis to authenticate the two tested ginsengs.	2002	42
SCAR	Two *Panax *species and adulterants	polymorphic RAPD fragment	There is a 25 bp insertion in *P. ginseng *for a polymorphic RAPD fragment. By using primers designed based on this polymorphic fragment, *P. quinquefolius *and *P. ginseng *can be differentiated. Amplification on four other *Panax *species and two ginseng adulterants gave markedly different products.	2001	91
ARMS	Five *Panax *species and corresponding Ginseng drugs	*trnK *and nuclear 18S gene sequences	Five sets of species-specific primers with two pairs in each set were designed. Two expected fragments, one from *trnK *gene and another from 18S rRNA gene, were observed simultaneously only when the set of species-specific primers encountered template DNA of the corresponding species.	2004	121
SSR	Two *Panax *species	primer sequences for the loci	Using nine of the 16 screened loci, Chinese ginseng was differentiated unambiguously from the American samples. Some of the informative loci showed different allelic patterns among ginsengs from different farms.	2003	25
SSR	American ginseng and Oriental ginseng, cultivated and wild American ginseng	primer sequences for the loci	American ginseng had a different allele pattern (allele frequency in an American ginseng population of 34 cultivated and 21 wild ginseng) in two microsatellite loci compared with that of the Oriental ginseng (six from China and South Korea). Cultivated and wild American ginseng were distinguished.	2005	141
Sequencing	Three *Panax *species	primer sequences for 18S region	The sequences of the 18S rRNA region of 3 *Panax *species have different base substitutions at four nucleotide positions. By the polymorphism, the ginseng species from commercial samples were identified.	1996	186
Sequencing	Twelve *Panax *species	primer sequences for ITS and 5.8S regions	The sequences of the ITS and 5.8S coding region were used to reconstruct the phylogenetic relationships among twelve *Panax *species. *P. quinquefolius *was suggested to be more closely related to the eastern Asian species than *P. trifolius*. Previous suggestions about monophyly of *P. ginseng, P. notoginseng *and *P. quinquefolius *were not supported by the ITS data. Several biogeographical implications were inferred from the results.	1996	187
Sequencing	*Panax notoginseng *of different cultivar origins	primer sequences for 18S and matK genes	The nuclear 18S rRNA and chloroplast *matK *genes of 18 samples of *Panax notoginseng *and its processed material *Sanqi *(*Radix Notoginseng*) were found to be identical regardless of cultivar origin. Thus the same strain should be used in cultivation for conservation of the species.	2006	188
Sequencing	*P. notoginseng *and adulterants	primer sequences for 18S and matK genes	*Notoginseng *and its adulterants were differentiated on the basis of their sequence variation in the nuclear ribosomal RNA small subunit (18S rRNA) and chloroplast *matK *gene.	2001	67
Sequencing	Two *Panax *species and adulterants	primer sequences for ITS and *rbcL *genes	The nuclear ITS and chloroplast *rbcL *regions were amplified using universal primers and then sequenced using nested ginseng-specific primers. All the samples including five root samples, thirteen powder samples and six capsule samples were identified as American or Korean ginseng except a capsule and a tablet that were identified as soybean, by using the universal primers for sequencing. They could not be sequenced by the nested primers.	2000	174

The results obtained from *Panax *do not represent Chinese medicines. The results of specific techniques depend on their conditions (e.g. primers and magnesium concentration).

## Conclusion

Authentication of Chinese medicinal materials is important for ensuring safe and appropriate use of Chinese medicines, ensuring the therapeutic effectiveness, minimizing unfair trade and raising consumers' confidence towards Chinese medicines. It also plays an important role in the modernization, industrialization and internationalization of Chinese medicine. DNA methods are reliable approaches towards authentication of Chinese medicinal materials. While their abilities in identification at various levels (e.g. species, strain and locality levels) may vary, they are used in identification of samples in any physical forms and provide consistent results irrespective of age, tissue origin, physiological conditions, environmental factors, harvest, storage and processing methods of the samples. The low requirement of sample quantity for analysis is of particular importance for quality control of premium medicinal materials and for detecting contaminants. For future development, it is necessary to compile a reference library of Chinese medicines with genetic information, especially for endangered species and those with high market value and/or with possible poisonous adulterants [243].

## Abbreviations

5S spacer 5S rRNA gene spacer

18S gene a gene encoding for the small ribosomal subunit

26S gene a gene encoding for the large ribosomal subunit

AFLP amplified fragment length polymorphism

AP-PCR arbitrarily primed PCR

ARMS amplification refractory mutation system

AS-PCR allele-specific PCR

atpβ gene encoding the β-subunit of ATP synthase

atpβ-*rbcL *intergenic spacer the spacer region between the *atpβ *and *rbcL *genes

BSA bovine serum albumin

*cyt b cytochrome b *gene

DAF DNA amplification fingerprinting

DALP direct amplification of length polymorphism

DAMD directed amplification of minisatellite-region DNA

ISSR inter-simple sequence repeat

ITS internal transcribed spacer

*matK *gene encoding a putative maturase for splicing the precursor of *trnK*

*ndhF *gene encoding the ND5 protein of chloroplast NADH dehydrogenase

PCR polymerase chain reaction

PCR-RFLP PCR-restriction fragment length polymorphism

RAPD random amplified polymorphic DNA

*rbcL *gene encoding large subunit of ribulose-1,5-bisphosphate carboxylase/oxygenase

rDNA ribosomal DNA

RP-PCR random-primed PCR

rRNA genes ribosomal RNA genes

SCAR sequence characterized amplified regions

SSLP simple sequence length polymorphism

SSR simple sequence repeat

STMS sequence-tagged microsatellite sites

*trnK *chloroplast transfer RNA (tRNA) gene for lysine

*trnL *– *trnF *spacer non-coding intergenic spacer between *trnL *and *trnF *in the chloroplast genome

*trnL *intron intron region between the two exons of tRNA gene for leucine

VNTR variable number of tandem repeat

## Competing interests

The author(s) declare that they have no competing interests.

## Authors' contributions

PYY drafted the manuscript. CFC, CYM and HSK helped draft and revised the manuscript. All authors read and approved the final manuscript.
